# Poly[tetra­kis(2,2′-bipyridine)undeca-μ-oxido-hexa­oxidodicopper(II)hexa­vanadium(V)]

**DOI:** 10.1107/S1600536810014224

**Published:** 2010-04-24

**Authors:** Ji-Wen Cui, Xiao-Bing Cui, Hai-Hui Yu, Ji-Qing Xu, Shu-Hong Wang

**Affiliations:** a College of Chemistry and Pharmacy, Jiamusi University, Jiamusi 154000, People’s Republic of China; b State Key Laboratory of Inorganic Synthesis and Preparative Chemistry, College of Chemistry, Jilin University, Changchun 130021, People’s Republic of China; c College of Chemical Engineering, Northeast Dianli University, Jilin 132000, People’s Republic of China

## Abstract

In the title organic–inorganic hybrid vanadate complex, [Cu_2_V_6_O_17_(C_10_H_8_N_2_)_4_]_*n*_, the Cu^II^ atom is six-coordinated by two chelating 2,2′-bipyridine (bipy) ligands and two vanadate O atoms in a distorted octa­hedral geometry. Two [Cu(bipy)_2_V_3_O_8_] units are linked by a bridging O atom, which lies on an inversion center, forming a dimeric unit. The dimeric units are further connected by bridging vanadate O atoms into a two-dimensional layer parallel to (100). The layers are connected by weak C—H⋯O hydrogen bonds.

## Related literature

For the introduction of some transition metal complexes into inorganic framework structures, see: Cao *et al.* (2003 ▶); Liu *et al.* (2001 ▶); Zhang *et al.* (2000 ▶).
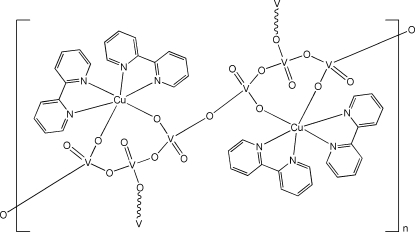


## Experimental

### 

#### Crystal data

[Cu_2_V_6_O_17_(C_10_H_8_N_2_)_4_]
*M* *_r_* = 1329.46
Monoclinic, 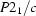

*a* = 15.512 (3) Å
*b* = 14.761 (3) Å
*c* = 10.470 (2) Å
β = 92.00 (3)°
*V* = 2395.9 (8) Å^3^
*Z* = 2
Mo *K*α radiation
μ = 2.07 mm^−1^
*T* = 293 K
0.57 × 0.40 × 0.30 mm

#### Data collection

Rigaku R-AXIS RAPID diffractometer
Absorption correction: multi-scan (*ABSCOR*; Higashi, 1995 ▶) *T*_min_ = 0.385, *T*_max_ = 0.577
20154 measured reflections
4742 independent reflections
4010 reflections with *I* > 2σ(*I*)
*R*_int_ = 0.027

#### Refinement

*R*[*F* ^2^ > 2σ(*F* ^2^)] = 0.031
*wR*(*F* ^2^) = 0.087
*S* = 1.05
4742 reflections
395 parameters
All H-atom parameters refined
Δρ_max_ = 0.47 e Å^−3^
Δρ_min_ = −0.67 e Å^−3^

### 

Data collection: *RAPID-AUTO* (Rigaku, 1998 ▶); cell refinement: *RAPID-AUTO*; data reduction: *RAPID-AUTO*; program(s) used to solve structure: *SHELXS97* (Sheldrick, 2008 ▶); program(s) used to refine structure: *SHELXL97* (Sheldrick, 2008 ▶); molecular graphics: *ORTEPIII* (Burnett & Johnson, 1996 ▶); software used to prepare material for publication: *SHELXL97*.

## Supplementary Material

Crystal structure: contains datablocks I, global. DOI: 10.1107/S1600536810014224/hy2298sup1.cif

Structure factors: contains datablocks I. DOI: 10.1107/S1600536810014224/hy2298Isup2.hkl

Additional supplementary materials:  crystallographic information; 3D view; checkCIF report

## Figures and Tables

**Table 1:** Hydrogen-bond geometry (Å, °)

*D*—H⋯*A*	*D*—H	H⋯*A*	*D*⋯*A*	*D*—H⋯*A*
C11—H8⋯O1	0.87 (3)	2.52 (3)	3.093 (4)	124 (2)
C14—H5⋯O5^i^	0.89 (4)	2.51 (3)	3.159 (5)	131 (3)
C18—H3⋯O5^ii^	0.90 (4)	2.46 (4)	3.315 (5)	157 (3)
C19—H2⋯O9^iii^	0.98 (4)	2.32 (4)	3.156 (5)	144 (3)

Symmetry codes: (i) 
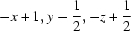
; (ii) 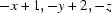; (iii) 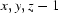.

**Fig. 1.**
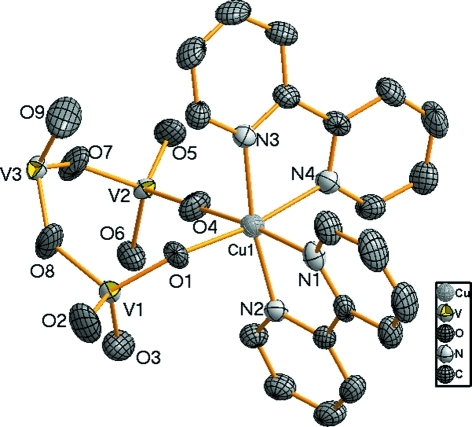
The asymmetric unit of the title compound, with displacement ellipsoids at the 50% probability level.

**Fig. 2.**
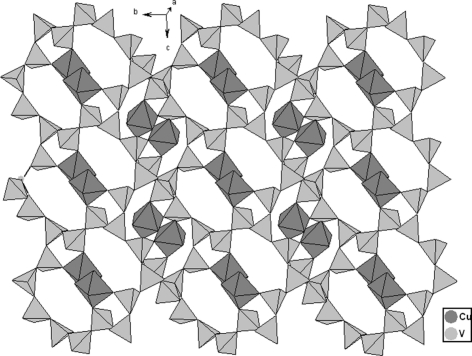
A polyhedral representation of the two-dimensional layer-like structure in the title compound. H and C atoms of bipy molecules are omitted for clarity.

## supplementary crystallographic information

### Comment

An important advance for the design of organic-inorganic hybrid materials is to introduce some transition metal complexes (TMCs) into the backbone of inorganic oxides (Liu *et al.*, 2001; Zhang *et al.*, 2000). We are interested in introducing transition metal complexes into inorganic frameworks and understanding the role of metal complexes on the modification of inorganic framework structures (Cao *et al.*, 2003). In an effort to further explore the structural diversity of the M/V/O/L system (M = transition metal, L = organic ligand), we have prepared the title compound.

The asymmetric unit of the title compound contains a [Cu(bipy)_2_(V_3_O_8.5_)] (bipy = 2,2'-bipyridine) unit, as shown in Fig.1. The V^V^ centers exhibit VO_4_ tetrahedral coordination environments with V—O_t_ (terminal O atom) distances ranging from 1.580 (3) to 1.610 (2) Å and V—O_b_ (bridging O atom) distances ranging from 1.630 (2) to 1.821 (2) Å. The Cu^II^ atom is six-coordinated by two bipy ligands and two vanadate O atoms (O1 and O4) in a distorted octahedral geometry, with Cu—N = 2.055 (2)–2.125 (2) Å and Cu—O = 2.025 (2) and 2.082 (2) Å. Two [Cu(bipy)_2_V_3_O_8_] units are linked by a bridging O2 atom, which lies on an inversion center, with a V—O distance of 1.7813 (6) Å, generating a dimeric [Cu_2_(bipy)_4_V_6_O_17_] unit. As illustrated in Fig. 2, each dimeric unit is joined to four adjacent ones through O6 and its symmetry equivalents, generating a two-dimensional network grafted with [Cu(bipy)_2_]^2+^ complex. In addition, the adjacent two-dimension layers further stack into a three-dimensional structure via weak C—H···O hydrogen-bonding interactions (Table 1).

### Experimental

A mixture of Na_2_WO_4_.2H_2_O (1.20 g, 3.6 mmol), V_2_O_5_ (0.33 g, 1.8 mmol), Cu(CH_3_CO_2_)_2_.4H_2_O (0.3 g, 1.2 mmol), bipy (0.18 g, 1.2 mmol) and distilled water (20 ml, 1111 mmol) in a molar ratio of 6:3:2:2:1850 was stirred for 120 min. The pH value of the mixture was necessarily adjusted to 4 with dilute H_3_PO_4_ solution. The resultant mixture was sealed in a 25 ml Teflon-lined autoclave and heated at 553 K for 72 h. The autoclave was then cooled to room temperature. The crystalline product was filtered, washed with distilled water and dried at ambient temperature to give 0.335 g solids of the title compound.

### Refinement

All H atoms were located from difference Fourier maps and refined isotropically.

### Crystal data

**Table:**

[Cu_2_V_6_O_17_(C_10_H_8_N_2_)_4_]	*F*(000) = 1320
*M**_r_* = 1329.46	*D*_x_ = 1.843 Mg m^−^^3^
Monoclinic, *P*2_1_/*c*	Mo *K*α radiation, λ = 0.71073 Å
Hall symbol: -P 2ybc	Cell parameters from 7302 reflections
*a* = 15.512 (3) Å	θ = 2.4–26.0°
*b* = 14.761 (3) Å	µ = 2.07 mm^−^^1^
*c* = 10.470 (2) Å	*T* = 293 K
β = 92.00 (3)°	Block, red
*V* = 2395.9 (8) Å^3^	0.57 × 0.40 × 0.30 mm
*Z* = 2	

### Data collection

**Table:**

Rigaku R-AXIS RAPID diffractometer	4742 independent reflections
Radiation source: fine-focus sealed tube	4010 reflections with *I* > 2σ(*I*)
graphite	*R*_int_ = 0.027
Detector resolution: 10 pixels mm^-1^	θ_max_ = 26.1°, θ_min_ = 2.4°
ω scans	*h* = −19→19
Absorption correction: multi-scan (*ABSCOR*; Higashi, 1995)	*k* = −18→18
*T*_min_ = 0.385, *T*_max_ = 0.577	*l* = −12→12
20154 measured reflections	

### Refinement

**Table:**

Refinement on *F*^2^	Primary atom site location: structure-invariant direct methods
Least-squares matrix: full	Secondary atom site location: difference Fourier map
*R*[*F*^2^ > 2σ(*F*^2^)] = 0.031	Hydrogen site location: inferred from neighbouring sites
*wR*(*F*^2^) = 0.087	All H-atom parameters refined
*S* = 1.05	*w* = 1/[σ^2^(*F*_o_^2^) + (0.0468*P*)^2^ + 1.8261*P*] where *P* = (*F*_o_^2^ + 2*F*_c_^2^)/3
4742 reflections	(Δ/σ)_max_ = 0.001
395 parameters	Δρ_max_ = 0.47 e Å^−^^3^
0 restraints	Δρ_min_ = −0.67 e Å^−^^3^

### Fractional atomic coordinates and isotropic or equivalent isotropic displacement parameters (Å^2^)

**Table:**

	*x*	*y*	*z*	*U*_iso_*/*U*_eq_	
Cu1	0.23318 (2)	0.97904 (2)	0.20082 (3)	0.02820 (11)	
V1	0.08531 (3)	1.05781 (3)	0.42475 (4)	0.02542 (12)	
V2	0.31303 (3)	1.19857 (3)	0.31446 (4)	0.02286 (12)	
V3	0.25041 (3)	1.14320 (3)	0.60425 (4)	0.02692 (12)	
O1	0.14691 (13)	0.98560 (13)	0.3484 (2)	0.0341 (5)	
O2	0.0000	1.0000	0.5000	0.0553 (10)	
O3	0.04662 (16)	1.13047 (15)	0.3236 (2)	0.0510 (6)	
O4	0.27773 (15)	1.10202 (14)	0.2580 (2)	0.0427 (5)	
O5	0.41332 (14)	1.21072 (16)	0.2836 (2)	0.0436 (5)	
O6	0.25003 (15)	1.28957 (15)	0.2414 (2)	0.0430 (5)	
O7	0.30503 (16)	1.20006 (18)	0.4860 (2)	0.0510 (6)	
O8	0.14570 (16)	1.12151 (18)	0.5470 (2)	0.0551 (7)	
O9	0.2978 (2)	1.05097 (19)	0.6372 (3)	0.0846 (11)	
N1	0.17768 (16)	0.86159 (15)	0.1249 (2)	0.0318 (5)	
N2	0.15122 (14)	1.03192 (15)	0.0616 (2)	0.0268 (5)	
N3	0.32645 (15)	0.91624 (15)	0.3159 (2)	0.0294 (5)	
N4	0.33465 (15)	0.96215 (17)	0.0718 (2)	0.0318 (5)	
C1	0.1889 (2)	0.7771 (2)	0.1683 (3)	0.0430 (8)	
C2	0.1533 (3)	0.7026 (2)	0.1050 (4)	0.0546 (11)	
C3	0.1052 (3)	0.7157 (2)	−0.0051 (4)	0.0527 (10)	
C4	0.0924 (2)	0.8026 (2)	−0.0504 (4)	0.0437 (8)	
C5	0.12893 (18)	0.87492 (19)	0.0171 (3)	0.0313 (6)	
C6	0.11711 (17)	0.97065 (19)	−0.0214 (3)	0.0292 (6)	
C7	0.0741 (2)	0.9974 (3)	−0.1329 (3)	0.0428 (8)	
C8	0.0654 (2)	1.0887 (3)	−0.1598 (3)	0.0453 (8)	
C9	0.0985 (2)	1.1508 (2)	−0.0744 (3)	0.0393 (7)	
C10	0.14073 (19)	1.1201 (2)	0.0355 (3)	0.0333 (6)	
C11	0.3215 (2)	0.9033 (2)	0.4409 (3)	0.0407 (7)	
C12	0.3903 (3)	0.8710 (3)	0.5148 (4)	0.0550 (10)	
C13	0.4660 (3)	0.8537 (3)	0.4584 (4)	0.0615 (12)	
C14	0.4726 (2)	0.8668 (3)	0.3293 (4)	0.0498 (9)	
C15	0.40065 (19)	0.8971 (2)	0.2594 (3)	0.0340 (7)	
C16	0.40090 (19)	0.9128 (2)	0.1201 (3)	0.0343 (6)	
C17	0.4643 (2)	0.8791 (3)	0.0428 (4)	0.0478 (9)	
C18	0.4617 (3)	0.8992 (3)	−0.0851 (4)	0.0562 (10)	
C19	0.3964 (2)	0.9526 (3)	−0.1333 (3)	0.0509 (9)	
C20	0.3348 (2)	0.9822 (3)	−0.0522 (3)	0.0409 (8)	
H1	0.299 (2)	1.013 (2)	−0.078 (3)	0.032 (9)*	
H2	0.387 (2)	0.969 (3)	−0.223 (4)	0.057 (11)*	
H3	0.508 (3)	0.880 (2)	−0.129 (4)	0.053 (11)*	
H4	0.498 (2)	0.846 (3)	0.078 (4)	0.050 (11)*	
H5	0.522 (3)	0.856 (2)	0.292 (3)	0.052 (11)*	
H6	0.510 (3)	0.835 (3)	0.498 (4)	0.065 (12)*	
H7	0.384 (3)	0.867 (3)	0.598 (4)	0.072 (13)*	
H8	0.272 (2)	0.916 (2)	0.474 (3)	0.030 (8)*	
H9	0.1628 (19)	1.159 (2)	0.094 (3)	0.026 (8)*	
H10	0.091 (2)	1.207 (3)	−0.088 (3)	0.052 (11)*	
H11	0.034 (3)	1.110 (3)	−0.245 (4)	0.070 (12)*	
H12	0.056 (2)	0.956 (3)	−0.177 (4)	0.050 (11)*	
H13	0.065 (3)	0.813 (3)	−0.128 (4)	0.072 (14)*	
H14	0.077 (3)	0.658 (3)	−0.049 (4)	0.077 (13)*	
H15	0.164 (3)	0.651 (3)	0.129 (4)	0.060 (12)*	
H16	0.223 (2)	0.772 (2)	0.247 (3)	0.040 (9)*	

### Atomic displacement parameters (Å^2^)

**Table:**

	*U*^11^	*U*^22^	*U*^33^	*U*^12^	*U*^13^	*U*^23^
Cu1	0.02821 (19)	0.02505 (19)	0.0313 (2)	0.00175 (13)	−0.00022 (14)	−0.00123 (13)
V1	0.0201 (2)	0.0241 (2)	0.0322 (3)	−0.00464 (17)	0.00256 (18)	−0.00629 (18)
V2	0.0239 (2)	0.0238 (2)	0.0208 (2)	−0.00305 (17)	−0.00113 (17)	0.00312 (17)
V3	0.0344 (3)	0.0247 (2)	0.0215 (2)	0.00049 (19)	−0.00231 (19)	−0.00010 (18)
O1	0.0324 (11)	0.0333 (11)	0.0370 (11)	0.0057 (9)	0.0089 (9)	−0.0015 (9)
O2	0.0417 (19)	0.0462 (19)	0.080 (3)	−0.0205 (16)	0.0305 (18)	−0.0156 (18)
O3	0.0606 (16)	0.0355 (12)	0.0561 (15)	0.0119 (11)	−0.0094 (12)	−0.0029 (11)
O4	0.0535 (14)	0.0283 (11)	0.0450 (13)	−0.0079 (10)	−0.0142 (11)	−0.0008 (9)
O5	0.0289 (11)	0.0584 (15)	0.0438 (13)	−0.0057 (10)	0.0052 (9)	0.0031 (11)
O6	0.0523 (14)	0.0443 (13)	0.0329 (11)	0.0190 (11)	0.0073 (10)	0.0107 (10)
O7	0.0553 (15)	0.0756 (17)	0.0221 (11)	−0.0188 (13)	0.0007 (10)	0.0027 (11)
O8	0.0452 (14)	0.0773 (18)	0.0430 (14)	−0.0242 (13)	0.0016 (11)	−0.0233 (12)
O9	0.140 (3)	0.0501 (16)	0.0618 (18)	0.0471 (18)	−0.0262 (19)	−0.0005 (14)
N1	0.0354 (14)	0.0246 (12)	0.0361 (14)	−0.0028 (10)	0.0089 (11)	−0.0038 (10)
N2	0.0231 (12)	0.0260 (12)	0.0312 (12)	0.0005 (9)	−0.0015 (9)	−0.0020 (9)
N3	0.0325 (13)	0.0271 (12)	0.0286 (12)	0.0054 (10)	−0.0012 (10)	−0.0004 (9)
N4	0.0277 (13)	0.0359 (13)	0.0317 (13)	0.0023 (10)	0.0020 (10)	0.0009 (10)
C1	0.053 (2)	0.0291 (16)	0.047 (2)	−0.0013 (14)	0.0123 (17)	−0.0002 (14)
C2	0.074 (3)	0.0237 (17)	0.068 (3)	−0.0073 (17)	0.027 (2)	−0.0034 (16)
C3	0.060 (2)	0.0374 (19)	0.061 (2)	−0.0164 (17)	0.0191 (19)	−0.0196 (17)
C4	0.0414 (19)	0.0415 (19)	0.048 (2)	−0.0090 (15)	0.0062 (16)	−0.0190 (15)
C5	0.0275 (15)	0.0319 (15)	0.0350 (16)	−0.0018 (12)	0.0076 (12)	−0.0102 (12)
C6	0.0220 (13)	0.0353 (15)	0.0303 (15)	−0.0013 (11)	0.0019 (11)	−0.0073 (12)
C7	0.0378 (18)	0.055 (2)	0.0354 (18)	−0.0037 (16)	−0.0039 (14)	−0.0108 (16)
C8	0.0395 (19)	0.059 (2)	0.0368 (18)	0.0058 (16)	−0.0055 (14)	0.0090 (16)
C9	0.0369 (18)	0.0387 (18)	0.0423 (18)	0.0035 (14)	0.0011 (14)	0.0102 (14)
C10	0.0311 (16)	0.0285 (15)	0.0399 (17)	0.0003 (12)	−0.0034 (13)	0.0004 (13)
C11	0.047 (2)	0.0427 (18)	0.0322 (17)	0.0091 (15)	−0.0002 (15)	−0.0034 (14)
C12	0.076 (3)	0.056 (2)	0.0317 (19)	0.014 (2)	−0.0137 (18)	0.0014 (16)
C13	0.060 (3)	0.067 (3)	0.055 (2)	0.025 (2)	−0.026 (2)	−0.0006 (19)
C14	0.039 (2)	0.056 (2)	0.054 (2)	0.0190 (17)	−0.0066 (17)	−0.0005 (17)
C15	0.0329 (16)	0.0292 (15)	0.0397 (17)	0.0067 (12)	−0.0023 (13)	−0.0009 (12)
C16	0.0293 (15)	0.0335 (15)	0.0401 (17)	0.0057 (12)	0.0023 (13)	0.0003 (13)
C17	0.042 (2)	0.050 (2)	0.052 (2)	0.0176 (17)	0.0068 (17)	−0.0025 (17)
C18	0.057 (2)	0.063 (2)	0.050 (2)	0.0111 (19)	0.0220 (19)	−0.0076 (18)
C19	0.054 (2)	0.065 (2)	0.0344 (19)	0.0032 (18)	0.0077 (16)	0.0014 (17)
C20	0.0333 (18)	0.053 (2)	0.0360 (18)	0.0042 (16)	−0.0004 (14)	0.0054 (15)

### Geometric parameters (Å, °)

**Table:**

Cu1—O4	2.025 (2)	C2—H15	0.82 (4)
Cu1—N2	2.055 (2)	C3—C4	1.380 (5)
Cu1—N3	2.069 (2)	C3—H14	1.06 (4)
Cu1—N1	2.081 (2)	C4—C5	1.390 (4)
Cu1—O1	2.082 (2)	C4—H13	0.91 (4)
Cu1—N4	2.125 (2)	C5—C6	1.479 (4)
V1—O3	1.609 (2)	C6—C7	1.382 (5)
V1—O1	1.656 (2)	C7—C8	1.382 (5)
V1—O2	1.7813 (6)	C7—H12	0.82 (4)
V1—O8	1.821 (2)	C8—C9	1.367 (5)
V2—O5	1.610 (2)	C8—H11	1.05 (4)
V2—O4	1.630 (2)	C9—C10	1.380 (4)
V2—O7	1.805 (2)	C9—H10	0.85 (4)
V2—O6	1.814 (2)	C10—H9	0.90 (3)
V3—O9	1.580 (3)	C11—C12	1.381 (5)
V3—O7	1.740 (2)	C11—H8	0.87 (3)
V3—O8	1.741 (2)	C12—C13	1.358 (6)
V3—O6^i^	1.746 (2)	C12—H7	0.88 (4)
O2—V1^ii^	1.7813 (6)	C13—C14	1.373 (6)
O6—V3^iii^	1.746 (2)	C13—H6	0.83 (4)
N1—C1	1.336 (4)	C14—C15	1.388 (4)
N1—C5	1.351 (4)	C14—H5	0.89 (4)
N2—C10	1.338 (4)	C15—C16	1.477 (4)
N2—C6	1.349 (3)	C16—C17	1.387 (4)
N3—C11	1.328 (4)	C17—C18	1.370 (5)
N3—C15	1.342 (4)	C17—H4	0.79 (4)
N4—C20	1.332 (4)	C18—C19	1.366 (5)
N4—C16	1.344 (4)	C18—H3	0.90 (4)
C1—C2	1.389 (5)	C19—C20	1.372 (5)
C1—H16	0.97 (3)	C19—H2	0.98 (4)
C2—C3	1.365 (6)	C20—H1	0.77 (3)
			
O4—Cu1—N2	93.75 (9)	C1—C2—H15	121 (3)
O4—Cu1—N3	90.30 (9)	C2—C3—C4	119.3 (3)
N2—Cu1—N3	170.39 (9)	C2—C3—H14	117 (2)
O4—Cu1—N1	172.68 (9)	C4—C3—H14	123 (2)
N2—Cu1—N1	78.93 (10)	C3—C4—C5	119.2 (4)
N3—Cu1—N1	96.93 (10)	C3—C4—H13	121 (3)
O4—Cu1—O1	87.74 (9)	C5—C4—H13	120 (3)
N2—Cu1—O1	96.38 (9)	N1—C5—C4	121.3 (3)
N3—Cu1—O1	92.48 (9)	N1—C5—C6	115.3 (2)
N1—Cu1—O1	93.10 (9)	C4—C5—C6	123.4 (3)
O4—Cu1—N4	92.31 (10)	N2—C6—C7	121.3 (3)
N2—Cu1—N4	92.71 (9)	N2—C6—C5	115.0 (2)
N3—Cu1—N4	78.41 (9)	C7—C6—C5	123.7 (3)
N1—Cu1—N4	88.01 (9)	C6—C7—C8	119.5 (3)
O1—Cu1—N4	170.89 (9)	C6—C7—H12	114 (3)
O3—V1—O1	108.66 (12)	C8—C7—H12	126 (3)
O3—V1—O2	110.17 (10)	C9—C8—C7	119.2 (3)
O1—V1—O2	110.81 (8)	C9—C8—H11	120 (2)
O3—V1—O8	106.82 (13)	C7—C8—H11	121 (2)
O1—V1—O8	112.34 (12)	C8—C9—C10	118.8 (3)
O2—V1—O8	107.96 (8)	C8—C9—H10	120 (3)
O5—V2—O4	109.82 (12)	C10—C9—H10	121 (3)
O5—V2—O7	107.40 (12)	N2—C10—C9	122.7 (3)
O4—V2—O7	109.75 (11)	N2—C10—H9	116.5 (19)
O5—V2—O6	110.05 (11)	C9—C10—H9	120.8 (19)
O4—V2—O6	108.97 (11)	N3—C11—C12	122.1 (3)
O7—V2—O6	110.83 (11)	N3—C11—H8	116 (2)
O9—V3—O7	109.65 (17)	C12—C11—H8	122 (2)
O9—V3—O8	109.78 (17)	C13—C12—C11	119.0 (4)
O7—V3—O8	108.38 (12)	C13—C12—H7	123 (3)
O9—V3—O6^i^	108.97 (14)	C11—C12—H7	118 (3)
O7—V3—O6^i^	109.07 (11)	C12—C13—C14	119.9 (3)
O8—V3—O6^i^	110.97 (12)	C12—C13—H6	124 (3)
V1—O1—Cu1	141.73 (12)	C14—C13—H6	116 (3)
V1^ii^—O2—V1	180.00 (3)	C13—C14—C15	118.6 (4)
V2—O4—Cu1	175.90 (14)	C13—C14—H5	120 (2)
V3^iii^—O6—V2	138.61 (13)	C15—C14—H5	121 (2)
V3—O7—V2	138.95 (15)	N3—C15—C14	121.5 (3)
V3—O8—V1	141.98 (16)	N3—C15—C16	115.7 (3)
C1—N1—C5	118.9 (3)	C14—C15—C16	122.8 (3)
C1—N1—Cu1	126.9 (2)	N4—C16—C17	121.5 (3)
C5—N1—Cu1	114.19 (18)	N4—C16—C15	115.3 (3)
C10—N2—C6	118.6 (3)	C17—C16—C15	123.1 (3)
C10—N2—Cu1	125.6 (2)	C18—C17—C16	119.7 (3)
C6—N2—Cu1	114.90 (18)	C18—C17—H4	125 (3)
C11—N3—C15	119.0 (3)	C16—C17—H4	115 (3)
C11—N3—Cu1	125.1 (2)	C19—C18—C17	118.8 (3)
C15—N3—Cu1	115.52 (19)	C19—C18—H3	125 (2)
C20—N4—C16	117.4 (3)	C17—C18—H3	115 (2)
C20—N4—Cu1	128.3 (2)	C18—C19—C20	118.6 (3)
C16—N4—Cu1	113.47 (19)	C18—C19—H2	126 (2)
N1—C1—C2	122.1 (4)	C20—C19—H2	115 (2)
N1—C1—H16	116 (2)	N4—C20—C19	123.9 (3)
C2—C1—H16	122 (2)	N4—C20—H1	117 (3)
C3—C2—C1	119.2 (4)	C19—C20—H1	119 (3)
C3—C2—H15	119 (3)		
			
O3—V1—O1—Cu1	36.1 (2)	N1—Cu1—N4—C16	−90.4 (2)
O2—V1—O1—Cu1	157.26 (16)	C5—N1—C1—C2	1.1 (5)
O8—V1—O1—Cu1	−81.9 (2)	Cu1—N1—C1—C2	−175.5 (3)
O4—Cu1—O1—V1	37.4 (2)	N1—C1—C2—C3	0.0 (6)
N2—Cu1—O1—V1	−56.2 (2)	C1—C2—C3—C4	−0.6 (6)
N3—Cu1—O1—V1	127.6 (2)	C2—C3—C4—C5	0.1 (5)
N1—Cu1—O1—V1	−135.4 (2)	C1—N1—C5—C4	−1.6 (4)
O5—V2—O6—V3^iii^	33.7 (3)	Cu1—N1—C5—C4	175.4 (2)
O4—V2—O6—V3^iii^	−86.8 (2)	C1—N1—C5—C6	177.8 (3)
O7—V2—O6—V3^iii^	152.3 (2)	Cu1—N1—C5—C6	−5.3 (3)
O9—V3—O7—V2	81.2 (3)	C3—C4—C5—N1	1.0 (5)
O8—V3—O7—V2	−38.6 (3)	C3—C4—C5—C6	−178.3 (3)
O6^i^—V3—O7—V2	−159.5 (2)	C10—N2—C6—C7	1.7 (4)
O5—V2—O7—V3	−138.5 (2)	Cu1—N2—C6—C7	−167.8 (2)
O4—V2—O7—V3	−19.1 (3)	C10—N2—C6—C5	−177.9 (2)
O6—V2—O7—V3	101.3 (3)	Cu1—N2—C6—C5	12.6 (3)
O9—V3—O8—V1	−54.8 (3)	N1—C5—C6—N2	−4.8 (4)
O7—V3—O8—V1	64.9 (3)	C4—C5—C6—N2	174.6 (3)
O6^i^—V3—O8—V1	−175.4 (2)	N1—C5—C6—C7	175.7 (3)
O3—V1—O8—V3	−109.6 (3)	C4—C5—C6—C7	−5.0 (5)
O1—V1—O8—V3	9.4 (3)	N2—C6—C7—C8	−0.4 (5)
O2—V1—O8—V3	131.9 (2)	C5—C6—C7—C8	179.1 (3)
N2—Cu1—N1—C1	−174.2 (3)	C6—C7—C8—C9	−0.9 (5)
N3—Cu1—N1—C1	14.6 (3)	C7—C8—C9—C10	0.8 (5)
O1—Cu1—N1—C1	−78.3 (3)	C6—N2—C10—C9	−1.8 (4)
N4—Cu1—N1—C1	92.6 (3)	Cu1—N2—C10—C9	166.5 (2)
N2—Cu1—N1—C5	9.10 (19)	C8—C9—C10—N2	0.6 (5)
N3—Cu1—N1—C5	−162.12 (19)	C15—N3—C11—C12	0.1 (5)
O1—Cu1—N1—C5	105.0 (2)	Cu1—N3—C11—C12	−171.9 (3)
N4—Cu1—N1—C5	−84.0 (2)	N3—C11—C12—C13	1.4 (6)
O4—Cu1—N2—C10	−0.6 (2)	C11—C12—C13—C14	−1.2 (6)
N1—Cu1—N2—C10	179.4 (3)	C12—C13—C14—C15	−0.4 (6)
O1—Cu1—N2—C10	87.5 (2)	C11—N3—C15—C14	−1.9 (5)
N4—Cu1—N2—C10	−93.1 (2)	Cu1—N3—C15—C14	170.9 (3)
O4—Cu1—N2—C6	168.1 (2)	C11—N3—C15—C16	179.7 (3)
N1—Cu1—N2—C6	−11.84 (19)	Cu1—N3—C15—C16	−7.5 (3)
O1—Cu1—N2—C6	−103.8 (2)	C13—C14—C15—N3	2.0 (5)
N4—Cu1—N2—C6	75.6 (2)	C13—C14—C15—C16	−179.6 (3)
O4—Cu1—N3—C11	80.5 (3)	C20—N4—C16—C17	−3.9 (5)
N1—Cu1—N3—C11	−100.6 (3)	Cu1—N4—C16—C17	166.7 (3)
O1—Cu1—N3—C11	−7.2 (3)	C20—N4—C16—C15	176.4 (3)
N4—Cu1—N3—C11	172.8 (3)	Cu1—N4—C16—C15	−13.0 (3)
O4—Cu1—N3—C15	−91.7 (2)	N3—C15—C16—N4	13.9 (4)
N1—Cu1—N3—C15	87.1 (2)	C14—C15—C16—N4	−164.5 (3)
O1—Cu1—N3—C15	−179.5 (2)	N3—C15—C16—C17	−165.8 (3)
N4—Cu1—N3—C15	0.6 (2)	C14—C15—C16—C17	15.8 (5)
O4—Cu1—N4—C20	−93.8 (3)	N4—C16—C17—C18	2.6 (5)
N2—Cu1—N4—C20	0.1 (3)	C15—C16—C17—C18	−177.8 (3)
N3—Cu1—N4—C20	176.4 (3)	C16—C17—C18—C19	0.2 (6)
N1—Cu1—N4—C20	78.9 (3)	C17—C18—C19—C20	−1.5 (6)
O4—Cu1—N4—C16	96.9 (2)	C16—N4—C20—C19	2.6 (5)
N2—Cu1—N4—C16	−169.2 (2)	Cu1—N4—C20—C19	−166.4 (3)
N3—Cu1—N4—C16	7.1 (2)	C18—C19—C20—N4	0.1 (6)

Symmetry codes: (i) *x*, −*y*+5/2, *z*+1/2; (ii) −*x*, −*y*+2, −*z*+1; (iii) *x*, −*y*+5/2, *z*−1/2.

### Hydrogen-bond geometry (Å, °)

**Table:**

*D*—H···*A*	*D*—H	H···*A*	*D*···*A*	*D*—H···*A*
C11—H8···O1	0.87 (3)	2.52 (3)	3.093 (4)	124 (2)
C14—H5···O5^iv^	0.89 (4)	2.51 (3)	3.159 (5)	131 (3)
C18—H3···O5^v^	0.90 (4)	2.46 (4)	3.315 (5)	157 (3)
C19—H2···O9^vi^	0.98 (4)	2.32 (4)	3.156 (5)	144 (3)

Symmetry codes: (iv) −*x*+1, *y*−1/2, −*z*+1/2; (v) −*x*+1, −*y*+2, −*z*; (vi) *x*, *y*, *z*−1.
